# Public understanding of local tornado characteristics and perceived protection from land-surface features in Tennessee, USA

**DOI:** 10.1371/journal.pone.0219897

**Published:** 2019-07-24

**Authors:** Kelsey N. Ellis, Lisa Reyes Mason, Kelly N. Gassert

**Affiliations:** 1 Department of Geography, University of Tennessee, Knoxville, Tennessee, United States of America; 2 College of Social Work, University of Tennessee, Knoxville, Tennessee, United States of America; Cardiff University, UNITED KINGDOM

## Abstract

Misunderstandings about the influence of land-surface features on tornado frequency and other tornado-related misconceptions may affect how people prepare for and behave during hazardous weather events. This research uses a phone survey (*n* = 1804) to assess how participants in three regions of Tennessee perceive their local tornado characteristics (*i.e*., direction of travel, seasonality, and diurnal timing) and their belief in protection from land-surface features (*i.e*., hills, water bodies, and buildings). Region of residence influences most beliefs in local tornado characteristics, and demographic characteristics, specifically age and gender, also have some influence. Residents in hilly East Tennessee are more likely to believe they are protected by hills and underestimate the proportion of nocturnal tornadoes, while residents in West Tennessee are more likely to believe they are protected by water bodies, perhaps because of proximity to the Mississippi River. Outside of the typical severe-weather season, participants were uncertain of when tornadoes were likely to occur; specifically, they did not recognize their local wintertime tornado activity. Because public perceptions are related to local features, local organizations and personnel, for example National Weather Service offices and broadcast meteorologists, may be most helpful in dispelling these misconceptions.

## Introduction

Recent devastating tornado outbreaks in the southeast United States (SEUS), for example, the April 2011 outbreak which produced 25 killer tornadoes [1], have heightened the need to better understand physical and social vulnerability to tornadoes in the region. After the April 2011 outbreak, researchers found that residents based their decisions during the event, in part, on their knowledge and beliefs about their local risk and vulnerability to tornadoes [2]. Many of these beliefs provided a false sense of security and may have put residents in danger. In this study, we examine public knowledge and beliefs about climatological tornado characteristics across the state of Tennessee. Through improved understanding about the extent of these conceptions, research can potentially inform strategies for dispelling misconceptions that may exist.

Tennessee is in the SEUS, a region where the active tornado season lasts longer than the non-tornado season. In addition to an active spring, over 20% of historical tornadoes in the SEUS have occurred both in the winter (Dec-Jan-Feb) and fall (Sep-Oct-Nov) [3]. A climatological minimum of tornadoes has occurred in the summer (Jun-Jul-Aug), which can be considered the short “off-season.” Fuhrmann et al. [4] showed that tornadoes not associated with outbreaks occurred at an almost even rate across the year in the SEUS, much different than other regions of the United States, which had clear springtime peaks. Additionally, while all regions in the US had a peak in outbreak tornadoes in the spring, the SEUS had a clear second peak in the fall [4]. The proportion of tornadoes occurring during the cold season (Nov-Dec-Jan-Feb) in Tennessee increased significantly between 1953 and 2015 [5], while having decreased in other locations. These cold-season tornadoes provide forecasting and communication challenges because people may be distracted by the holiday season and forecasters may be presented with a more challenging forecasting environment [6].

Daily timing is also an important, unique part of the SEUS tornado climatology. The SEUS is home to the greatest proportion of nocturnal tornadoes in the US, and in Tennessee nearly half of tornadoes between 1950 and 2005 occurred at night [7]. This is especially important because nocturnal tornadoes are more likely to cause a fatality than those occurring during the day [7]. Not only are nocturnal tornadoes hard to detect because of additional forecasting challenges at night [8] and a lack of visibility [7], but the public is often asleep, possibly in weak structures, increasing their vulnerability [7].

Understanding characteristics of the local tornado climatology may affect how people prepare for and respond to a tornado event. Prior to an event, risk perception, including the perceived likelihood of an event, may influence decisions such as whether to buy a home with a storm shelter [9]. During an event, these perceptions may affect a person’s attention paid to the potential threat. Mason et al. [10] showed that individuals who believed they have a low climatological tornado risk in their county said that they were less likely to receive a tornado warning at night, if a warning was issued. Even if the warning is communicated to them, a person may respond differently based on their perceived risk. As Lindell and Perry [11] discussed, an accurate threat perception corresponds to appropriate disaster response, specifically an individual’s decision on whether to take protective action. Thus, the likelihood of someone receiving a message, responding to it appropriately, and having a safe place to go may rely on their climatological knowledge.

In addition to a different tornado climatology in the SEUS, the physical and social settings of tornadoes in the region differ, affecting the vulnerability of residents. Tornadoes are especially challenging to spot in the SEUS because visibility is often blocked by hills and trees, darkness, or the rain of a high-precipitation supercell. Also, residents may develop perceptions of tornado risk related to their physical environment that may cause them to not respond safely to a tornado warning. A prime example is the belief that hills provide protection from tornadoes [2]. The relationship between terrain and tornadoes is not completely understood, although recent research suggests that fewer tornadoes may happen in areas with more terrain variability in the Great Plains [12]. Regardless of a terrain-tornado relationship, it is undoubtedly unsafe to assume that hills will provide protection from a pre-existing tornado. While not necessarily unique to the SEUS, similar beliefs exist surrounding the effects that rivers, highways, and other physical features [2] have on tornado behavior, when in reality these features will do nothing to protect someone in a tornado’s path. Social vulnerability also stems from high mobile-home density, poverty incidence, and population density of older adults [13]. These factors have contributed to a bullseye of historical tornado fatalities in southwest Tennessee that spreads to the northwest and southeast [13]. Over time this fatality maximum has been shifting southeast into Mississippi and Alabama [14].

We aim to assess Tennessee residents’ understanding of their region’s tornado characteristics. Residents from the three regions of the state were asked via phone survey about the seasonality, direction of travel, and diurnal timing of tornadoes; tornado threat relative to other regions of Tennessee (west, middle, and east); and perceived protection provided by hills, water bodies, and buildings. We compare survey responses to climatological data, and assess relationships between beliefs, geography, and demographic characteristics. We expect to find differences between the three regions of Tennessee because of the great variability in tornado climatology across the state, notably the lessened tornado frequency in East Tennessee [15], and the proximity to various land-surface types that may influence public perceptions, for example, the Smoky Mountains in East Tennessee and Mississippi River in West Tennessee.

## Data and methods

### Study area

**Fig 1:**
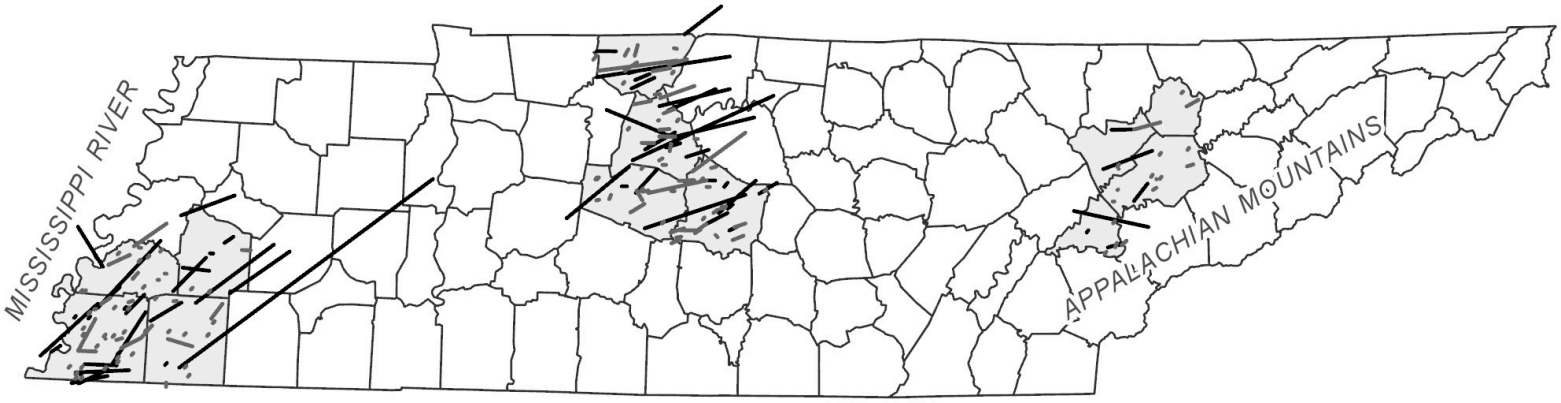
Study area. Counties used for the study are shown in grey. Tornadoes that affected those counties from 1965–2014 are shown in black (significant tornadoes; EF2 or greater) and grey (EF0 and EF1).

We sampled residents in four counties each of West, Middle, and East Tennessee. Sampling in only select counties allowed for more dense sampling in each region, rather than spreading our samples across the state, but does leave a large portion of the state unmeasured. In West Tennessee, we sampled Fayette, Haywood, Shelby (including the city of Memphis) and Tipton counties. In Middle Tennessee, we sampled Davidson (including the city of Nashville), Robertson, Rutherford, and Williamson counties. In East Tennessee, we sampled Anderson, Knox (including the city of Knoxville), Loudon, and Union counties. These counties, and the tornadoes that have occurred within them over a 50-year period, are shown in Fig 1. The population densities in each county vary greatly, and each region has a densely populated county that contains the city center (ranging from 528.5–755.3 persons per km^2^) and a more rural county with the lowest population density of the region (ranging from 21.9–86.5 persons per km^2^). The Middle Tennessee counties are the most populated on average.

### Tornado data

Tornado characteristics were gathered from the tornado database maintained by the Storm Prediction Center (SPC). The SPC database contains information for tornadoes observed since 1954, including the location (start and end points), date and time, intensity, and associated injuries and fatalities. We selected tornadoes that occurred within or intersected the selected counties (Fig 1) over a 50-year period (1965–2014) and calculated climatological characteristics including the monthly distribution of tornado occurrence, the proportion of tornadoes occurring at night, and the distribution of the direction of travel (for those tornado records that include track information). Because East Tennessee counties have such a small number of historical observations, the characteristics were averaged for each of the three regions (west, middle, or east) across their respective four counties for the analyses. There are known issues with the tornado database, most of which are not related to the variables included here. One issue with the data that may affect this work is the known population bias in the historical tornado record [16], as areas with lower population densities are more likely to “miss” spotting or observation of a tornado compared to heavily populated areas.

### Survey data

Surveys and interviews are a common method for obtaining information about public knowledge and perception of a natural hazard [17]. In tornado research, surveys and interviews have been used, for example, to gain information about decision-making in hypothetical/future tornado events [18, 19], past responses to a tornado warning [20–22], how the local environment influences risk perception [2], and the effect of prior experience on behavior during future events [23]. We use surveys in this study to obtain a relatively large, cost-effective sample size, as compared to other methods, for example, focus groups or interviews. We present our findings as representing those responding to the survey, not the entirety of the population in the study area. Because we only sampled select counties in each region, our results may not reflect the overall population of the three regions.

The surveys were completed by the Human Dimensions Research Laboratory at the University of Tennessee. Approval for the survey was obtained from the University of Tennessee Institutional Review Board for research with human subjects (UTK IRB-15-02696-XP). Participants were selected randomly from a list of cell phone and land line numbers from the 12 counties. Participants were 18 years or older, and informed consent was obtained from them before the survey began. They were asked 51 questions during the phone call, which lasted on average about 14 minutes. They received a ten-dollar (USD) gift card for their time. Participants were asked about their socioeconomic status, perceptions of tornado characteristics, and their intended behavior during tornadic events. Questions relevant to this study are listed in Table 1. There were 131–175 participants per county, for a total of 1804 participants. As is common in phone survey research, there were more female participants (63%) than male, and the sample represents a somewhat older portion of the population, with 34% of participants being 65 or older. More details about the sample population, counties, and survey questions are in Ellis et al. [24]. The surveys took place between February and July 2016. There may be a small seasonal bias to the responses based on when they were surveyed relative to any severe weather activity. Survey responses necessary for replication are available online at http://doi.org/10.3886/E109964V1.

**Table 1:** Survey questions pertaining to tornado experience, tornado climatology, and other tornado characteristics. Response options are given, though participants could also respond “I do not know” and some participants gave a non-listed response.

Question	Response options
Has a tornado ever hit your home?	Yes or No
Has a tornado ever hit a building while you were inside?	Yes or No
Has a tornado ever hit near where you live?	Yes or No
If 10 tornadoes hit _____ County in the upcoming years, how many of these would you expect to occur at night when it is dark?	Number 0–10
In which month or months would you say tornadoes are most likely to occur in ______ County?	Named month(s)
In which month or months would you say tornadoes are least likely to occur in ______ County?	Named month(s)
Which region of Tennessee do you think is most likely to be hit by a tornado?	West, Middle, or East
Which region do you think is least likely to be hit?	West, Middle, or East
If a tornado were to hit your area, which direction would the tornado most likely come from?	Named cardinal direction
To what extent do you think hills protect nearby places from tornadoes, if at all?	Not at all, Somewhat, Very much, Completely
To what extent do you think bodies of water, such as rivers and lakes, protect nearby places from tornadoes, if at all?	Not at all, Somewhat, Very much, Completely
To what extent do you think tall buildings protect nearby places from tornadoes, if at all?	Not at all, Somewhat, Very much, Completely

### Analyses

Descriptive statistics were used to assess participant understanding of tornado characteristics measured in the survey (Table 1) including: relative regional tornado likelihood, seasonality of tornadoes, daily timing of tornadoes, direction of tornado travel, and land-surface influences on tornadoes. Demographic variables used in the analyses included gender, age, and education. For education, participants selected from one of five categories: less than high school; high school graduate; some college, a technical or associate’s degree; or college graduate. Climatological values were calculated using the SPC tornado data from 1965–2014.

We selected several statistical tests to assess bivariate relationships. These models were selected prior to seeing any results (Table 2). Kruskal-Wallis tests were used if the dependent variable was ordinal and the independent variable categorical (ordinal or nominal). Thus, the test was used to determine the relationship between the perception of the proportion of tornadoes happening at night and the following independent variables: region, gender, education, prior experience, belief in protection from hills, belief in protection from water, and belief in the protection of buildings. Age was treated as a continuous independent variable and its effect on the perception of nocturnal tornado activity was assessed using logistic regression. These same tests were repeated to determine the relationship between the listed independent variables and each of the following dependent variables: belief in protection from hills, belief in protection from water, and belief in protection from buildings. Chi-square tests were used if both the independent and dependent variables were nominal; thus, they were used to determine how the region a respondent inhabits (independent variable) relates to their belief in which region is the most and least frequently hit by tornadoes (dependent variable).

**Table 2:** Variables used for analyses and their scale.

Variable	Scale
Region	Nominal
Gender	Nominal
Education	Ordinal
Age	Continuous
Prior experience	Ordinal
Perceived region of greatest likelihood	Nominal
Perceived region of lowest likelihood	Nominal
Belief in protection from hills	Ordinal
Belief in protection from trees	Ordinal
Belief in protection from buildings	Ordinal
Perceptions of nocturnal tornado activity	Ordinal

During the analysis we collapsed the categories for some variables in a logical manner to make the results easier to interpret. First, we collapsed participants’ prior experience into three categories. If a participant answered “yes” to the first or second question in Table 1, they were grouped together and are referred to as having the highest level of prior experience. It is logical to combine these two categories because both types of participants have been personally and directly impacted by a tornado, as the tornado hit their home or a building they were inside. A participant answering yes to the third question has the second-highest level of prior experience, as their experience with tornadoes was not necessarily direct. In the lowest level of prior experience were those who answered “no” to all three experience questions. This results in “direct experience,” “indirect experience,” and “no experience” categories. Prior experience was based solely on participant reports and was not fact checked, thus there could be issues of misreporting. Participant beliefs in the protection by hills, water bodies, and tall buildings were aggregated into three categories for each variable: belief in no protection (“not at all”), some protection (“somewhat”) or considerable protection (“very much” or “completely”). These categories were chosen because those participants that feel “very much” or “complete” protection from physical entities will likely make different decisions when responding to a tornado warning, ultimately affecting their personal safety. In each section where collapsed categories were used, we provide some discussion on the sensitivity of these decisions.

## Results and discussion

### Regional tornado frequencies

There was a large difference in historical tornado frequency across the state. During the 50-year study period, the most tornadoes were observed in the Middle Tennessee counties (105), but West Tennessee was close behind (99) and home to the county with the greatest number of tornadoes (Shelby; 50). The East Tennessee counties only experienced 30 total tornadoes. We can assume tornadoes were missed in all three regions, especially in areas with lower population densities. Based on these data, Middle and West Tennessee were the most likely to be hit by a tornado, with little difference between the two, and East Tennessee was the least likely.

Overall, 58% of participants selected the western region as most likely to be hit by a tornado, followed by the middle (28% of participants) and eastern (12% of participants) regions (Table 3). Within the western region, fewer participants from tornado-prone Shelby County (51% of participants) selected their region as having the greatest likelihood compared to participants from Haywood County (61%), which experienced the fewest tornadoes of the four western counties. There was more agreement when considering the region least likely to be hit by a tornado, with 71% of total participants selecting East Tennessee.

**Table 3:** Tennessee regions perceived as being the most and least likely to be hit by tornadoes.

**Most likely to be hit**			
	West	Middle	East
West participants	55.0	25.2	17.2
Middle participants	57.9	28.4	11.0
East participants	60.5	29.3	7.5
**Least likely to be hit**			
	West	Middle	East
West participants	17.7	16.4	62.4
Middle participants	10.8	12.9	73.2
East participants	9.1	10.2	77.1

Given is the % of participants from a given region who selected the listed region as the most or least likely to be hit by a tornado.

Chi-square results indicate a participant’s perception of regional tornado likelihood was not independent of their home region (Most likely: *χ*^2^ = 30.83, *p* < 0.01; Least likely: *χ*^2^ = 39.23, *p* < 0.01), meaning where they live affects where they think tornadoes are more likely to occur. This is supported by the descriptive statistics in Table 3, which suggests participants had different perceptions of their own region. More East Tennessee participants picked their home region as the least likely to be hit, showing they were aware of their relatively low tornado threat. West Tennessee had the highest percentage of “most likely to be hit” responses from residents in each of the three regions, suggesting that these participants may underestimate the heightened tornado threat in Middle Tennessee. Only 28.4% of Middle Tennessee participants named their region as the most likely to be hit. This could be concerning if it shapes perceptions of risk and vulnerability during tornado events, but it may not be that comparative likelihood affects decision-making. It could be that perceived likelihood of tornadoes influences planning, but not necessarily action, as Wirtz and Rohrbeck [25] found was the case for terrorism events.

A limitation to this work could be how recent memory plays a role in perceptions. For example, if a surveyed resident has not been affected by a tornado in recent time but they recall a friend or family member in a different region of the state being recently affected, they may tend to select the other region as having a higher likelihood of tornadoes. Similarly, if a place experiences intense tornadoes, people may perceive this place as being more at risk than a place that is actually hit more frequently, albeit with weaker tornadoes. This holds true for the remainder of the analyses; for example, if a recent significant tornado happened at night in March it may affect an individual’s perception of when tornadoes happen in their area, having them select the characteristics related to that specific event.

### Seasonality

**Fig 2:**
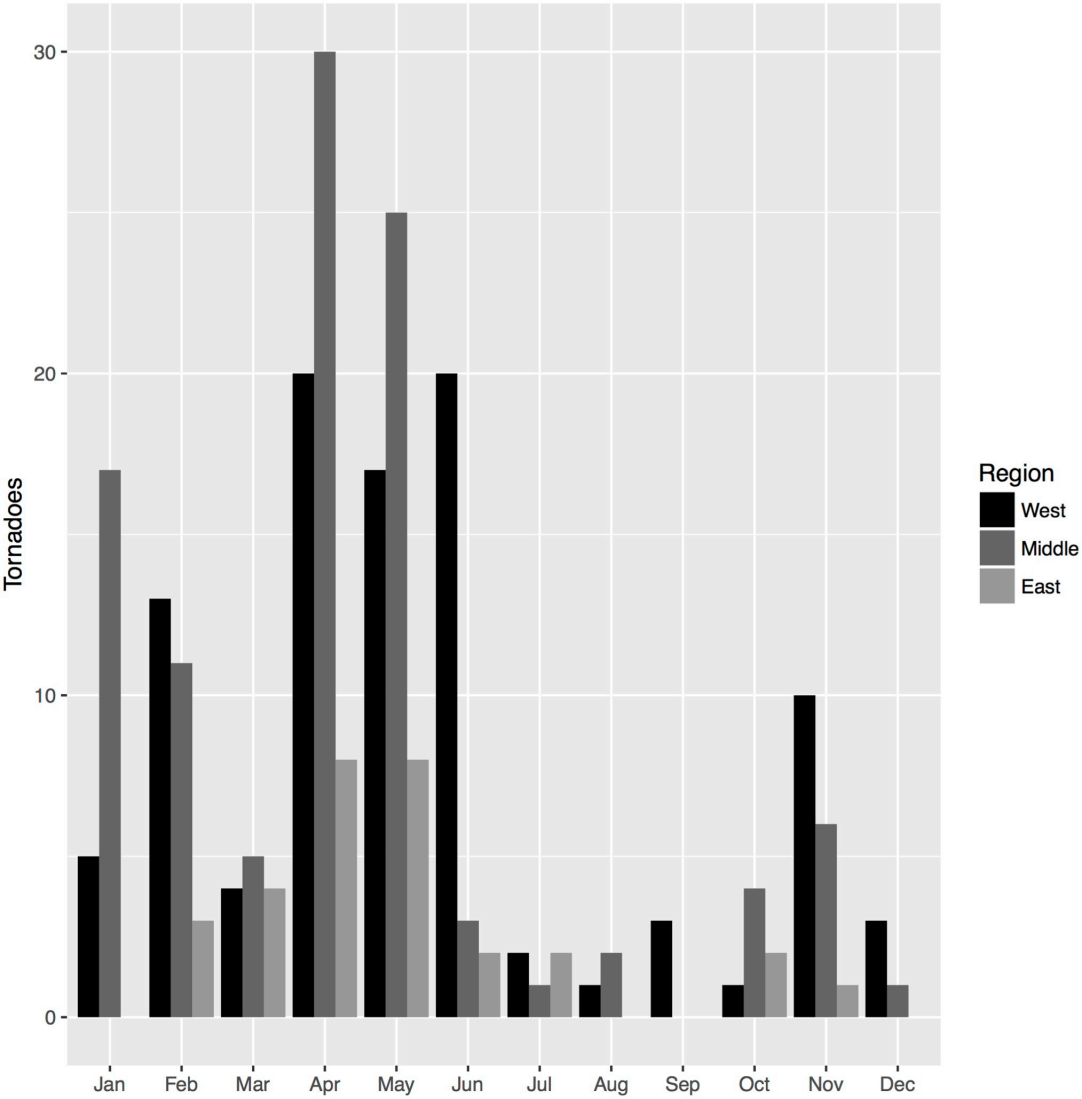
Tornado seasonality. Number of tornadoes that occurred each month during the study period in the twelve counties, aggregated by region.

The seasonality of tornadoes for each region is shown in Fig 2. Brown et al. [15] have previously shown these three areas experience peaks and minimums of activity at similar times of year. The most active month for all regions in the study period was April, although it was tied with May in the eastern region and June in the western region. Meanwhile, June was inactive in Middle and East Tennessee. Winter activity was evident in all regions, but more defined in West and Middle Tennessee. All regions showed a small winter peak in November, and larger peak in January–February. This second winter peak was less evident in East Tennessee where there was a smaller sample size, and February activity was more of a gradual introduction to the springtime peak.

Participants were asked to list the months tornadoes were most and least likely in their area, allowing us to gauge their perception of tornado seasonality. Some responses were descriptions, including three participants who indicated they think tornadoes can happen at any time, and a fourth who suggested tornadoes are more common when the season changes. Seventy-eight participants indicated they do not know what months are most active. Two responses to the question regarding the least active months also indicated tornadoes happen all year, and 52 participants indicated they do not know what months are least active.

**Fig 3:**
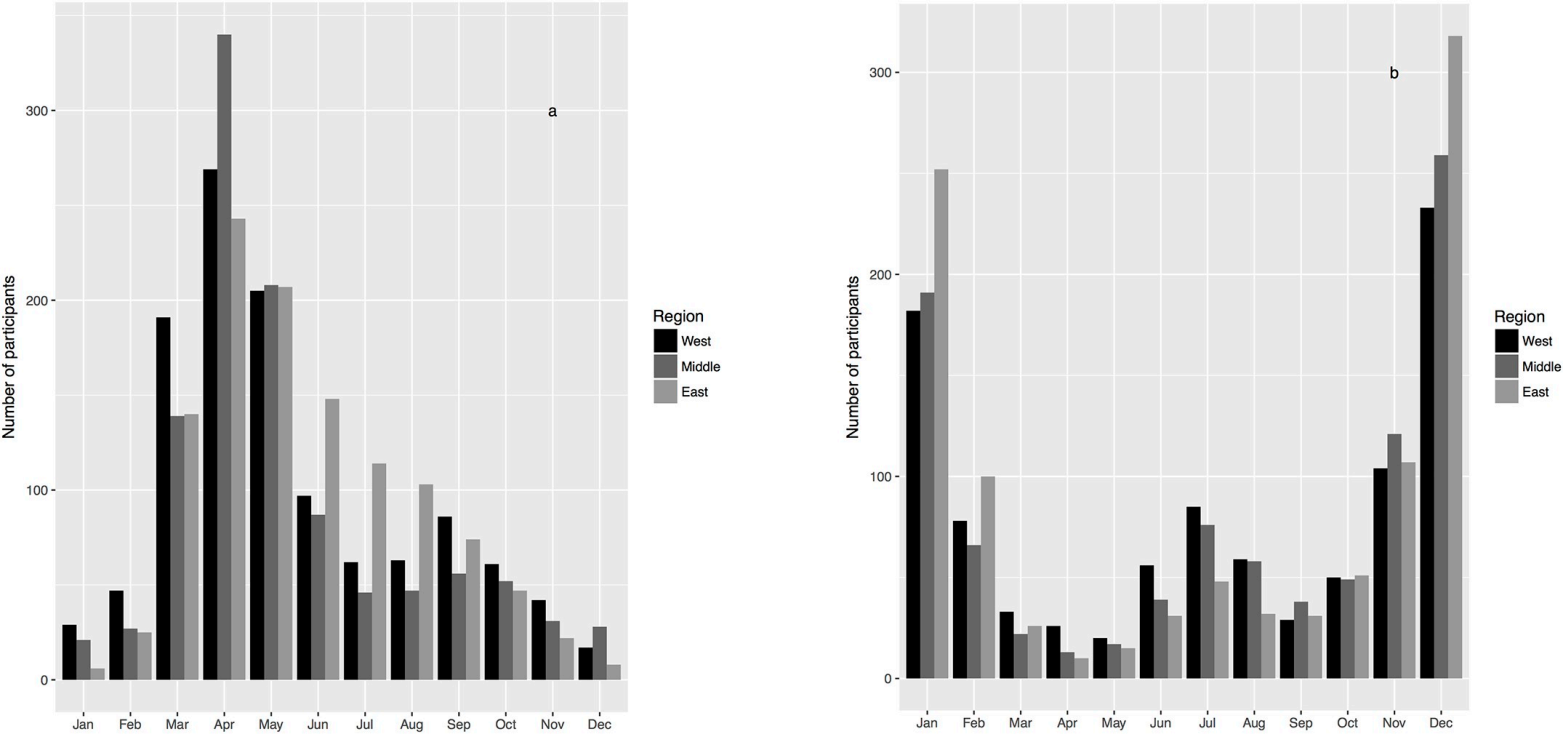
Perceived tornado seasonality. Number of participants who selected each month as the most (a) and least (b) active month for tornadoes in their county.

We aggregated the remaining answers by region (Fig 3) for a visual comparison to the regional climatological results in Fig 2. Participants could select multiple months. Of the participants that responded to the question regarding the most active months, the average number of months they selected was two. Nearly half of respondents selected one month (45%); 9% selected 4 or more months. Of the participants that responded to the question regarding the least active months, the average number of months they selected was also two. The majority of respondents selected one month (61%); 6% selected 4 or more months.

April, May, and March were selected most for being the most active months. Participants from all three regions followed this top-three order for most active months, except for in the eastern region where June replaced March as third most active. Overall, these results suggest participants recognized the typical spring tornado season, even if a month or two off.

There was disagreement among participants’ responses about the remainder of the warm season. Some residents thought tornadoes were likely in the other warmer months, including July–October. July and August were also selected by many as not being likely to host tornadoes, suggesting overall confusion about summer activity, especially in East Tennessee.

Participants did not recognize the heightened late fall/winter seasonal tornado activity. November was listed as the third least-likely tornado month, behind January and December, followed by February as fourth. Few participants selected a winter month as the most likely to have a tornado. In East Tennessee this is not surprising, since few tornadoes have occurred during the winter months in recent memory. Whether this indicates a lack of preparation for winter-time tornadoes in Tennessee or otherwise influences participant behavior during winter tornadoes is an important next question.

### Nocturnal tornadoes

The proportion of tornadoes that occurred at night was calculated for each region using the historical data. A tornado was considered nocturnal if the time of initiation fell between the sunset and sunrise time of the nearest major city (Memphis, Nashville, or Knoxville). During the study period, nearly half of the tornadoes occurred at night in each of the three regions; specifically, 45% of West Tennessee tornadoes, 50% of Middle Tennessee tornadoes, and 47% of East Tennessee tornadoes were nocturnal, comparable to the aforementioned findings of Ashley et al. [7].

Participants were asked if ten tornadoes were to occur, how many would happen at night. We chose ten tornadoes because it is an even, conceivable number of tornadoes, and could easily be turned into a percentage of expected nocturnal tornadoes. The largest proportion of participants in all three regions (27–29% per region) correctly estimated about 50% of tornadoes occur at night. The remaining responses were well dispersed among the other answers. It is encouraging that 50% was the most popular answer, suggesting most residents are aware nocturnal tornadoes regularly occur in their area; however, it is also possible that “about half” may have been more of a quick guess and not an accurate representation of the participants’ beliefs [26].

Not all participants recognized their nocturnal tornado threat. Ten percent or more of participants in each region said 0 or 10% of tornadoes occur nocturnally. A similar proportion of participants (approximately 10%) said 90 or 100% of tornadoes occur nocturnally. It would be meaningful to determine if such a low perception of nocturnal or daytime activity causes differential responses to tornado warnings based on time of day.

For the bivariate analyses, participants were grouped according to their perception of the proportion of tornado activity that happens at night. In the “low” nocturnal-activity group were those who estimated 0–2 of 10 tornadoes would occur at night, in the “moderate” nocturnal-activity group were those who estimated 3–7 of 10 tornadoes would occur at night, and in the “high” nocturnal-activity group were those who estimated 8–10 of 10 tornadoes would occur at night. The groups were selected to highlight those in either tail, where participants believed a large proportion of tornadoes occur during the day or at night.

Results indicated group membership (low, moderate, or high) is not identical across regions or participants with differing beliefs in the amount of protection provided by hills (Table 4). The details of this relationship can be discerned from the frequency table (Table 5). Participants from West Tennessee and those who believed they are considerably protected by hills during a tornado were more likely to believe a disproportionately large proportion of tornadoes occur at night. Participants from East Tennessee and those who believed they are not protected by hills at all during a tornado were more likely to believe a disproportionately small proportion of tornadoes occur at night. Results do not change if the independent belief variables (belief in the protection of hills, water, and buildings) are in the original four categories or the three collapsed categories. The findings demonstrate that understanding one characteristic of your local tornado threat (e.g., you are not protected by hills) does not necessarily indicate a strong overall understanding (e.g., about half occur at night).

**Table 4:** Results of bivariate analyses separately testing the relationship between one of the listed independent variables and one of the dependent variables (beliefs in relative nocturnal frequency, and protection provided by hills, water, and buildings).

Independent var.	Test	Nocturnal	Hills	Water	Buildings
Region	Kruskal-Wallis	9.63**	75.39**	13.99**	0.14
Gender	Kruskal-Wallis	1.93	0.48	5.44*	4.70*
Education	Kruskal-Wallis	1.28	2.37	5.16	6.51
Age	Logistic regression	0.99, 1.00	0.99	0.99**	1.00
Prior experience	Kruskal-Wallis	2.55	2.43	2.09	0.88
Hills	Kruskal-Wallis	6.42*	na	na	na
Water	Kruskal-Wallis	1.25	44.24**	na	na
Buildings	Kruskal-Wallis	0.51	24.39**	55.27**	na

For testing age as the independent variable, multinomial logistic regression was used when nocturnal activity perception was the dependent variable because the proportional odds assumption was not met; ordinal logistic regression was used for the remaining tests where age is the independent variable. For Kruskal-Wallis tests the *χ*^2^ value is given. Frequency information for the significant Kruskal-Wallis results for each dependent variable are in Tables 5, 6, 7 and 8. The odds ratio (OR) is given for the logistic regressions. For the multinomial logistic regression the first OR compares middle with lowest group. Significance values:

* *p* < 0.05,

** *p* < 0.01.

**Table 5:** Frequencies of group memberships (number of participants) for categories of the significant predictors of perceived proportion of nocturnal tornado activity. Significant predictors include region and perceived amount of protection provided by hills.

	Low proportion	Moderate proportion	High proportion
Region: West	102	321	122
Region: Middle	116	318	100
Region: East	143	306	97
Hills: No protection	61	150	54
Hills: Some protection	199	524	148
Hills: Considerable protection	97	259	114

Participants in the low proportion of nocturnal-activity group perceived 0–20% of tornadoes occurred at night, the moderate nocturnal-activity group perceived 30–70%, and the high nocturnal-activity group perceived 80–100% of tornadoes occurred at night.

### Direction of travel

**Fig 4:**
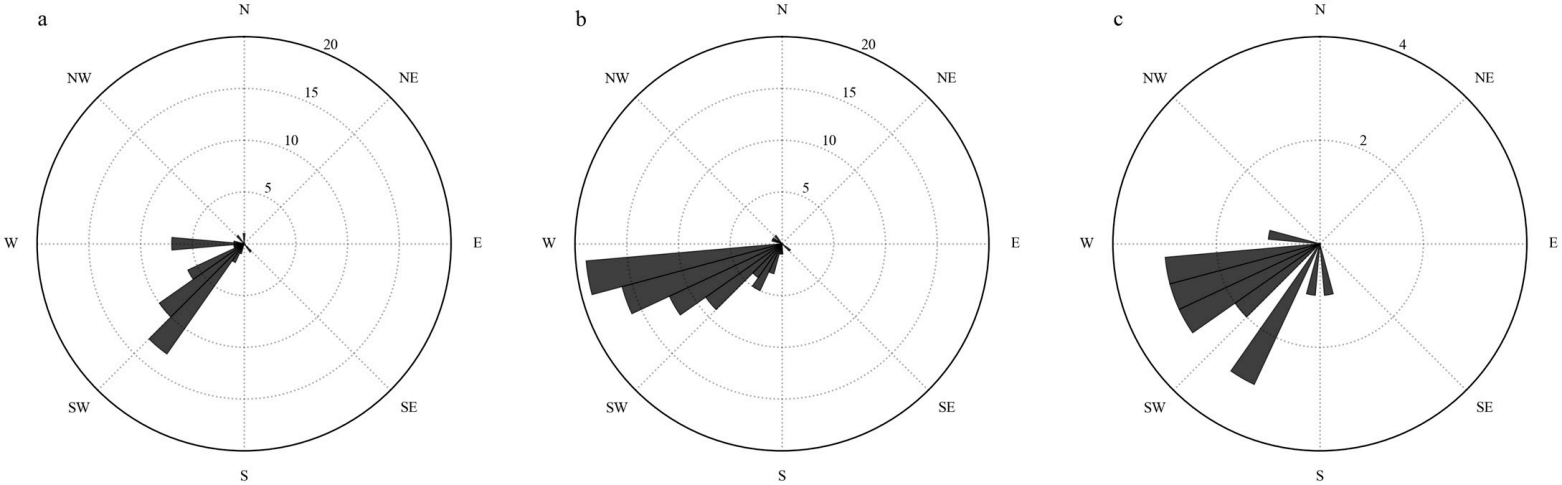
Direction of travel for tornadoes in the western (a), middle (b), and eastern (c) regions. Numbers indicate the number of tornadoes coming from the given direction.

Historical tornado paths were plotted on a polar grid to visualize direction of travel in each of the three regions of Tennessee (Fig 4). Direction of tornado travel is toward the center of each polar grid. These directions were calculated using the start and end locations from the SPC tornado database and EasyCalculate 10, an Add-in for ArcGIS 10.0 and above. Tornadoes with no recorded end location are not depicted. All three regions exhibited a predominately southwest to northeast direction of travel.

**Fig 5:**
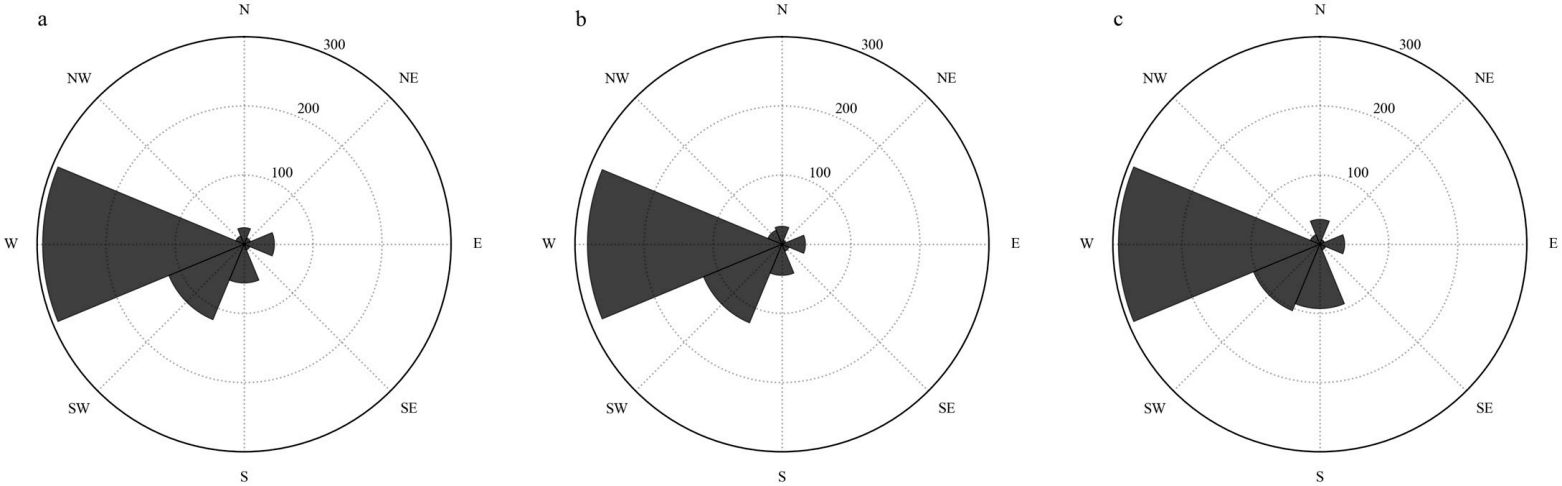
Perceived direction of travel for tornadoes in the western (a), middle (b), and eastern (c) regions. Numbers indicate the number of participants selecting the given direction.

Among participant perceptions of direction of travel (Fig 5), the majority recognized tornadic storms come from the west and southwest as they move across the state. West was the most prominent answer (48% of participants). Southwest, south, and west were the most popular answers for all three regions and account for 78% of the responses.

That the majority of participants understand the primary direction tornadoes travel in their area is a positive finding; however, it is important that their understanding of the climatology does not affect their response to a tornado outside of the climatological norm. For example, Schumacher et al. [27] found that a tornado in northern Colorado that traveled in an unusual direction and occurred at an unusual time of day was a challenging event for local decision makers to understand and communicate.

### Perceptions of protection from land-surface features

**Fig 6:**
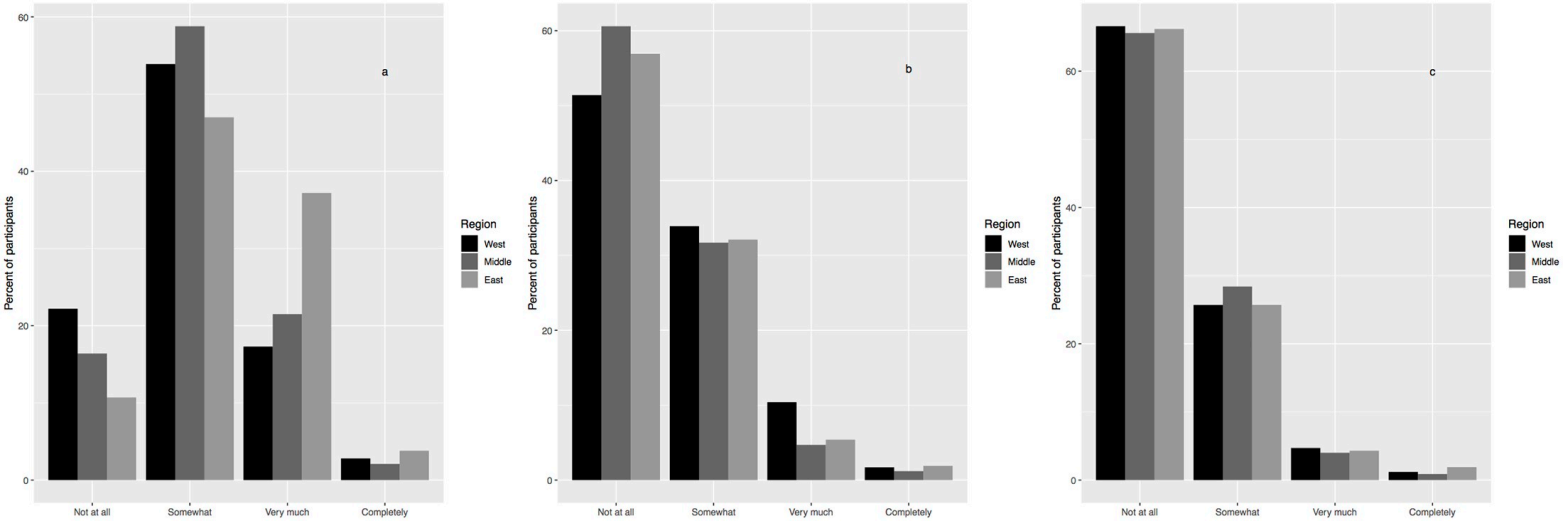
Participants’ belief in how much protection from tornadoes is provided by (a) hills, (b) water, and (c) buildings, given in % participants per region.

Among participant perceptions of how much land-surface features, specifically hills, water, and buildings, protect places from tornadoes (see Fig 6), the most prominent answer was that they were protected “somewhat” by hills (58%), and “not at all” by water (56%) and buildings (66%). East Tennessee participants were more likely to feel very or completely protected by hills, and West Tennessee participants were more likely to feel very or completely protected by water. No region stands out as having more or less belief in the protection from buildings.

Bivariate tests indicated that belief in the protection by hills and water by participants varied with region, while belief in the level of protection by buildings did not vary with region (Table 4). Specifically, the hilly terrain of East Tennessee may lead participants there to believe hills provide protection (Table 6). Meanwhile, West Tennessee participants may feel water provides some protection, perhaps because of their close proximity to the Mississippi River (Table 7). Gender played a role in the belief of protection by water and buildings, with male (female) participants more likely to believe in a higher level of protection from buildings (water bodies), as shown in Tables 7 and 8. Age may also play a role in the belief of protection by water; as age increases there is a slight decrease in this belief (Table 4). The three land-surface characteristic variables were not independent from each other, meaning, for example, belief in protection from hills is not independent from belief in protection from water. Results do not change if the dependent variables (belief in the protection of hills, water, and buildings) are in the original four categories or the three collapsed categories.

**Table 6:** Frequencies of group memberships (number of participants) for categories of the significant predictors of perceived amount of protection provided by hills. The predictors include region and perceived amount of protection provided by water and buildings.

	No protection	Some protection	Considerable protection
Region: West	133	323	127
Region: Middle	95	340	136
Region: East	67	295	257
Water: No protection	201	557	243
Water: Some protection	66	325	188
Water: Considerable protection	21	54	74
Buildings: No protection	226	640	308
Buildings: Some protection	55	261	157
Buildings: Considerable protection	12	45	43

**Table 7:** Frequencies of group memberships (number of participants) for categories of the significant predictors of perceived amount of protection provided by water. The predictors include region, gender, and perceived amount of protection provided by buildings.

	No protection	Some protection	Considerable protection
Region: West	308	203	72
Region: Middle	350	183	34
Region: East	357	201	46
Gender: Female	618	394	98
Gender: Male	394	188	54
Buildings: No Protection	729	181	41
Buildings: Some Protection	243	181	41
Buildings: Considerable Protection	30	45	24

**Table 8:** Frequencies of group memberships (number of participants) for gender and perceived amount of protection provided by buildings.

	No protection	Some protection	Considerable protection
Gender: Female	777	289	59
Gender: Male	411	188	42

Our regional results supported those of Klockow et al. [2], in that weather myths or “folk science” ideas surrounding tornado risk develop from connections to and experiences in local environments. These differences in regional beliefs could explain why our responses were different from those of Van Den Broeke and Arthurs [28], who found only 17% and 25% of participants correctly indicated rivers and cities, respectively, do not provide protection from tornadoes; our study participants indicated a better understanding of the lack of protection from water bodies (58%) and buildings (67%). Such different findings highlight the importance of individual experience and local knowledge for informing risk perception.

## Conclusion

We sampled residents in each of three Tennessee regions to assess their understanding of local tornado characteristics. The variation of tornado climatology across the state and other geographic differences between the regions were related to the participants’ views of their regional tornado characteristics. East Tennessee participants were more aware than residents of the other regions that tornado activity is greater in the western two-thirds of the state. Meanwhile, East Tennessee participants were less aware of the tornado seasonality, perhaps because they have a smaller sample size of tornadoes on which to base their knowledge.

Belief in the protection from local geographic features is also related to a participant’s home region, specifically their belief in the protection provided by hills and water bodies. More residents in East Tennessee believe they are protected by hills, perhaps because of the proximity of the sampled counties to the Smoky Mountains, while more residents in West Tennessee believe they are protected by water, perhaps because of the proximity of the sampled counties to the Mississippi River. This supports the conclusions of Klockow et al. [2] that knowledge and beliefs surrounding tornado risk and vulnerability are connected to a person’s geography and past experiences. These are also ideas that local meteorologists are used to hearing. When these results were presented at the 2018 Spring Partners Workshop in Memphis, some attendees commented on the frequency with which they heard these “folk” beliefs. Informing residents that rivers and hills will not protect them from a tornado headed their way is important, as Klockow et al. [2] showed these beliefs can affect a person’s actions during a tornado event.

The majority of participants from all regions recognized they experience a moderate amount of their tornadoes at night, but some residents believe a disproportionately large or small proportion of tornadoes occur at night. It is imperative that residents in the SEUS recognize their threat of nocturnal tornadoes, as they make up nearly half of the tornadoes in Tennessee, may require more planning, and are more likely to result in a fatality than tornadoes during daylight hours [7]. Recognizing their risk at night and listening to trusted sources is important for people’s safety at a time of day when information is sparse, environmental cues are negligible, and issued warnings are less likely to be received [10].

Tornado seasonality is also important for SEUS residents to understand, so they are prepared for tornado events outside of the traditional spring tornado season. Participants were confident tornadoes occur in the spring. There seemed to be a lack of understanding surrounding tornado activity during other warm months, and the secondary peak of tornadoes seen in Tennessee [15] and across the SEUS in the cooler months was not well known. This is concerning because if a resident believes tornadoes do not happen at a particular time it may affect their likelihood to receive or respond to a tornado warning, and fall (September-October-November) tornado activity has increased over time [3].

Demographic characteristics, specifically age and gender (Table 4), had some influence on how individuals perceived the protective capabilities of land-surface features. Similar to our previous work, which focused on how frequently residents believed their county is hit by a tornado [24], demographic characteristics were not as important as region for determining understanding of tornado characteristics. One surprising result from this work was prior experience with tornadoes did not affect participant understanding. Our previous work with this survey sample showed prior experience was the largest contributor to a participant’s perceived frequency of tornadoes in their county [24], and was also important in determining if a participant was likely to receive a tornado warning at night if issued [10] and if they seek appropriate shelter when receiving a warning [29], but here we have shown prior experience did not affect knowledge about the likelihood of nocturnal tornadoes, or whether one believes hills, water bodies, or buildings protect them from tornadoes.

Our results suggest a main contributor to a participant’s understanding of local tornado characteristics is their local geography, for example nearby land-surface features. Methods to improve public understanding of local tornado characteristics would require knowledge of local beliefs surrounding tornado activity, which vary across small areas, for example within a state. Local media markets and weather forecasting offices benefit from serving a specialized area and could be key partners in efforts to reduce the prevalence of some of the common misunderstandings of their audience.
